# Flow-Cytometric Monitoring of Minimal Residual Disease in Pediatric Patients With Acute Myeloid Leukemia: Recent Advances and Future Strategies

**DOI:** 10.3389/fped.2019.00412

**Published:** 2019-10-11

**Authors:** Barbara Buldini, Margarita Maurer-Granofszky, Elena Varotto, Michael N. Dworzak

**Affiliations:** ^1^Laboratory of Hematology-Oncology, Department of Woman's and Child's Health, University of Padova, Padova, Italy; ^2^Children's Cancer Research Institute (CCRI), St. Anna Kinderkrebsforschung, Vienna, Austria

**Keywords:** acute myeloid leukemia, minimal residual disease, multiparametric flow cytometry, childhood, risk stratification

## Abstract

Minimal residual disease (MRD) by multiparametric flow cytometry (MFC) has been recently shown as a strong and independent prognostic marker of relapse in pediatric AML (pedAML) when measured at specific time points during Induction and/or Consolidation therapy. Hence, MFC-MRD has the potential to refine the current strategies of pedAML risk stratification, traditionally based on the cytogenetic and molecular genetic aberrations at diagnosis. Consequently, it may guide the modulation of therapy intensity and clinical decision making. However, the use of non-standardized protocols, including different staining panels, analysis, and gating strategies, may hamper a broad implementation of MFC-MRD monitoring in clinical routine. Besides, the thresholds of MRD positivity still need to be validated in large, prospective and multi-center clinical studies, as well as optimal time points of MRD assessment during therapy, to better discriminate patients with different prognosis. In the present review, we summarize the most relevant findings on MFC-MRD testing in pedAML. We examine the clinical significance of MFC-MRD and the recent advances in its standardization, including innovative approaches with an automated analysis of MFC-MRD data. We also touch upon other technologies for MRD assessment in AML, such as quantitative genomic breakpoint PCR, current challenges and future strategies to enable full incorporation of MFC-MRD into clinical practice.

## Introduction

Pediatric acute myeloid leukemia (pedAML) is a heterogeneous group of hematological malignancies classified according to morphology, immunophenotyping, and genomics (1). In the last few decades, significant progress has been obtained in the outcome of pedAML, with an increase of survival rate up to 70% (2). The results depend on the improvement in risk stratification including genetic features at diagnosis, intensification of chemotherapy together with the amelioration of supportive care, and application of hematopoietic stem cell transplantation (HSCT) to specific subgroups of patients (3, 4). Nevertheless a large amount of patients (20–41%) encounters relapse, with non-acceptable final outcome in about 50–70% of them (5–13). Consequently, ever better definition of the factors predictive for relapse may improve the outcome. Among those, the residual disease assessment has been emerging as an ever more essential tool for patients' management and risk classification (6, 14–21).

Minimal or, more appropriately, measurable residual disease (MRD) detection allows the identification of 0.1–0.001% leukemic cells according to the adopted technique. MRD assessment in AML may establish a more in-depth remission status compared with the morphology-based evaluation, refining outcome prediction. A proper MRD approach should be sensitive and highly specific, reproducible and standardized with ideally an extended inter-institutional validation. Different techniques are currently available for MRD detection in pedAML, each one showing advantages and limitations: quantitative RNA-based polymerase chain reaction (RT-PCR) analysis of specific gene fusions, next-generation sequencing (NGS), gene expression profiling (GEP) and multiparametric flow cytometry detection (MFC) of aberrant immunophenotypes (22). Nowadays, RT-PCR and MFC-MRD are applied in the clinical setting (23, 24).

RT-PCR of fusion transcripts allows MRD detection with a sensitivity level of up to 0.001%. Regardless, it is applicable only in about 50–60% of pedAML with a detectable fusion gene or mutations. Moreover, a precise quantification of MRD may be difficult due to the unpredictable number of transcripts per leukemic cell (23). Among AML molecular alterations, the persistence of RUNX1-RUNX1T1 and CBFB-MYH11 fusion transcripts in continuous long-term remission have been already described, hampering the clinical relevance of MRD detection (25–27). Conversely, a slow molecular response at the end of Induction in t(8;21)-rearranged AML is associated with a higher risk of relapse when compared to an MRD reduction of at least 2 logs (28). Finally, different studies showed uncertain results on the role of FLT3-internal tandem duplication (ITD) and NPM1 mutations as MRD markers, with a potential instability of those lesions between diagnosis and relapse (14, 19). ITD-Allelic Ratio levels and molecular MRD should be included in the clinical management of FLT3-ITD AML patients since children with high levels of RT-PCR-MRD after the first Induction course had worse event free survival (EFS) (29).

NGS allows the identification of molecular anomalies, particularly relevant in AML with normal cytogenetics (30). In principle, it may be applied to all patients but requires highly specialized bioinformatics analysis. Moreover, the role of NGS in MRD detection is still controversial. While it may detect subclonal changes during therapy, potentially crucial for patients' outcome, NGS-MRD can be affected by mutations belonging to clonal hematopoiesis and ancestral clones (31).

MFC-MRD has a lower sensitivity than RT-PCR (up to 0.01%), but it is applicable in more than 90% of patients. Therefore, MFC-MRD is generally the method of choice for MRD detection in clinical AML studies. MFC-MRD may be assessed through two different techniques: (i) Leukemia-Associated Immunophenotype (LAIP) approach allows to identify leukemic blasts immunophenotype at diagnosis and track it at re-evaluation points; (ii) Different-from-Normal approach relies on the discrimination of aberrant immunophenotypes from normal cells during follow-up (32, 33).

This review focuses on the most relevant findings on MFC-MRD in pedAML and examines recent advances and new approaches for its standardization, including novel concepts for automated analysis of MFC-MRD data.

## MFC-MRD: From Past to Present

Even if MRD monitoring has become the standard of care in ALL, only recently it has been acquiring an even more important role in AML management (34).

In the last three decades, different retrospective studies on MFC-MRD monitoring have been performed in adult and pedAML. They suggested its strategic role in AML risk stratification. Regardless, to date, no guidelines or recommendations are available on methods, time points, and clinical application of MFC-MRD in AML. That depends on the heterogeneity of MRD assessment in AML, due to different available techniques, difficulties in comparing MRD data among laboratories and clinical trials, and the impact of sample quality on MRD level (34–38).

Regarding pediatric cohorts, in 2003, the COG group published the results of a prospective study on 252 *de novo* pediatric AMLs. MFC-MRD emerged as the most influential independent prognostic factor associated with poor outcome (39).

In the same year, Coustan-Smith et al. (14) applied to AML-MRD a four-color MFC approach usually adopted in pediatric acute lymphoblastic leukemia. That allowed to reach a sensitivity level of 0.1–0.01% of leukemic cells. MFC-MRD resulted as an independent predictor of outcome.

The described technique was subsequently applied to a cohort of 232 children consecutively enrolled in the AML02 multicenter trial. MFC-MRD was adopted as risk-stratification criteria together with the genetic features. MRD positivity was defined as 1 or more leukemic cells per 1,000 mononuclear bone marrow (BM) cells (≥0.1%). MRD positivity after Induction I was associated with an unfavorable outcome in high-risk AML (P = 0.01). Moreover, any MRD positivity after Induction II was predictive of an adverse outcome. The authors were able to monitor MRD in more than 90% of patients after each therapeutic course. The combined approach showed an improvement in patients' outcome (6).

In support to the St. Jude study, MFC-MRD was an independent prognostic variable in the Dutch Childhood Oncology Group ANLL 97/Medical Research Council of the UK AML12 experience, as well as in the COG AAML03P1 AML study (16, 18).

Regardless, the AML-BFM study published in 2006 did not find any significant role of MFC-MRD based on a standardized panel for four-color immunophenotyping in outcome prediction when compared to other known risk factors. A significant difference in 3-years EFS was demonstrated in the presence of positive MFC-MRD before the second Induction, and third therapy course but those data were not confirmed by a multivariable analysis including FAB subtype, cytogenetics, and morphologically determined blasts on day 15 (15).

Finally, two recent European studies strengthened the prognostic role of MFC-MRD monitoring in pedAML. In 2016, Tierens et al. (40) retrospectively analyzed MFC-MRD prognostic impact in a cohort of 201 children enrolled in the NOPHO-AML 2004 trial. MRD was detected by LAIP technique at two different time points (day 15 of Induction therapy and before Consolidation therapy). Samples with at least 0.1% leukemic events were considered MRD positive. In a univariate analysis, MFC-MRD positivity on day 15 and before Consolidation therapy was associated with a statistically significant adverse 5-years EFS and overall survival (OS). In a multivariate analysis including age, sex, leucocyte count, FLT3-ITD mutations, core-binding factor mutations, residual disease and BM morphology at both time points, only MFC-MRD positivity before Consolidation therapy still was associated with an unfavorable outcome, with a strong impact both on EFS and OS (40).

In 2017, Buldini et al. (41) published a retrospective study on the prognostic role of MFC-MRD in a cohort of 142 pedAML patients treated according to the Associazione Italiana di Emato-Oncologia Pediatrica (AIEOP)-AML 2002/01 trial. LAIP-MRD was assessed by 5-color MFC after the first and the second Induction courses, respectively, with a sensitivity cut-off of 0.1%. After the first Induction course, different MRD level (<0.1% vs. ≥ 0.1%) correlated with different 8-year disease-free survival (DFS) (73.1 ± 5.6% vs. 35.2 ± 7.2%, respectively, P < 0.01), as well as 8-years OS (82.2% vs. 51.6%, respectively, P < 0.01). Similar results were observed for MRD levels after the second Induction therapy course (8-years DFS: 68.4 ± 7·9% for MRD < 0.1% vs. 21.9 ± 9·4% for MRD ≥ 0.1%, P < 0.01; 8-years OS: 77.1% for MRD < 0.1% vs. 55.5% for MRD ≥0.1%). In a multivariate analysis, MRD ≥ 0.1% after the first Induction course was still associated with an adverse outcome (41).

Table 1 includes a complete list of the studies on MFC-MRD monitoring in AML.

**Table 1:** Principal studies on the role of MFC-MRD in childhood AML.

**References**	**Pts (n)**	**Age**	***De novo*/relapsed AML**	**Treatment protocol**	**BM evaluation time points**	**MRD cut-off**	**End point**
Sievers et al. (39)	252	<22	*De novo*	CCG-2941 CCG-2961	After I1 After I2 After C After Int	0.5%	3-year RFS 3-year OS
Coustan-Smith et al. (14)	54	<22	*De novo*	St. Jude AML97	After I1 After I2	0.1%	2-year OS 2-year LFS
Langebrake et al. (15)	150	0.06–20	*De novo*	AML BFM 98	Day 15 Before I2 Before TC3	Depending on LAIP specificity and evaluation time-point	3-year EFS 3-year FFS
van der Velden et al. (16)	94	1–16	*De novo*	MRC AML12 DCOG ANLL97	After TC1 After TC2 After TC3 EoT	<0.01% 0.01–0.1% 0.1–1% >1%	3-year RFS 3-year OS
Rubnitz et al. (6)	216	<21	*De novo*	AML02	After I1 After I2	<0.1% 0.1–1% >1%	3-year EFS 3-year CIR
Loken et al. (18)	249	<21	*De novo*	COG AAML03P1	EoI1 EoI2 EoT	EoI1: <0.1% 0.1–1% 1–5% >5% EoI2/EoT: RD pos/neg	3-year OS 3-year RFS 3-year RR
Inaba et al. (24)	203	<21	*De novo*	AML02	After I1 After I2	<0.1% 0.1–1% >1%	5-year EFS
Walter et al. (42)	253	any	*De novo*/relapsed	/	Before HCT	MRD neg <0.1% 0.1–1% >1%	3-year OS 3-year DFS 3-year NRM 3-year relapse
Karol et al. (20)	208	<21	*De novo*	AML02	After I1 After I2	0.1%	5-year EFS 5-year OS
Tierens et al. (40)	101	<18	*De novo*	NOPHO AML 2004	Day 15 Before C	0.1%	5-year EFS 5-year OS
Buldini et al. (41)	142	0.1–17.8	*De novo*	AIEOP AML 2002/01	After I1 After I2	0.1%	8-years OS 8-year EFS 8-year DFS 8-year CIR
Coustan-Smith et al. (43)	37	0.5–17	*De novo*	Malaysia-Singapore AML 2006	/	0.1%	/

*Pts, patients; n, number; AML, acute myeloid leukemia; BM, bone marrow; MRD, minimal residual disease; I1, Induction 1; I2, Induction 2; C, Consolidation; Int, Intensification; RFS, relapse-free survival; OS, overall survival; LFS, leukemia free survival; TC, therapy course; FFS, failure-free survival; EoT, End of Treatment; CIR, cumulative incidence of relapse; EoI, end of induction; RD, residual disease; pos, positive; neg, negative; RR, relapse risk; HSCT, hematopoietic stem cell transplantation; DFS, disease free survival; NRM, non-relapse mortality*.

## Challenges of MFC-MRD Assessment in AML

One of the most significant challenges of reliable MFC-MRD monitoring in AML is the requirement of well-trained experts in data interpretation. It is well recognized that intra- and inter-leukemic immunophenotypic heterogeneity is a common finding in AML (44). Leukemic blasts also need to be discriminated from normal myeloid progenitors during hematopoietic regeneration. Furthermore, immunophenotypic changes may occur during therapy, making MRD data interpretation even more challenging. Hence, a profound knowledge of myeloid cell compartments at various differentiation stages in normal and regenerating BM is required. To this aim, multicenter trials should contemplate an extensive training of MFC-MRD operators, including face-to-face activities with experts and internet-based data reviewing. Furthermore, a program for continuous quality control through recurrent ring trials is warranted. A ring trial is a proficiency testing in which identical samples are sent to the participating laboratories. The laboratories are expected to analyze the samples within an agreed period and send the results back to the coordinator center. That allows monitoring the performance of participating laboratories during time.

Besides, an extensive database of “normal” and regenerating BM at different time points aids in establishing a “range of normality” for each marker and immunophenotype. That can be achieved only by building up international networks among centralized reference laboratories to share resources and improve the applicability and accuracy of MFC-MRD (45).

## Standardization Efforts for MFC-MRD in AML

To ensure comparability of results among different laboratories, and facilitate the clinical use of MFC-MRD as a surrogate for OS and EFS, a standardized and reproducible assay is required (46, 47). Currently, multiple approaches for MFC-MRD detection and quantification are adopted: those refer to methodologies of sample processing, as well as antibody panels. There is no consensus on instrument set-up, data acquisition, analysis (e.g., gating strategies), and interpretation. The calculation of MRD load is not standardized yet, since different laboratories use different denominators for MRD enumeration (e.g., percentage of total nucleated cells, CD45^+^ cells, or mononuclear cells).

Several national and international networks have been established to optimize and standardize MFC-MRD detection and quantification (46–51). In this context, the European Leukemia Network recently published an extensive consensus document (52) on MFC-MRD measurement in adult AML with recommendations for common approaches including definition of time-points, thresholds, technical requirements, marker panels, and results reporting (32). Consistent adherence to such a standardized approach will likely overcome many of the current MFC-MRD limitations. Regardless, differences between pediatric and adult AML need to be considered, especially concerning LAIPs specificity (11, 53–55).

Conventional approaches for analysis and interpretation of MFC-MRD data, lag behind standardization of the wet-lab issues because of the many limitations of manual gating and data analysis strategies, especially when dealing with higher-dimensional complex datasets. Hence, data analysis and interpretation currently is kind of a bottleneck for safely applying MFC-MRD methodology in AML since it strongly relies on the operator's skills and is highly subjective and time-consuming (56). This is especially true for increasingly extensive marker combinations used for MRD detection. Several approaches for automated MFC data analysis have been proposed (57–64) to overcome this bottleneck by (i) providing a superior resolution compared to conventional manual gating with the whole multi-parameter MFC data space at once (instead of 2-D plot-based visualization), (ii) increasing results comparability and reproducibility through reduction of subjectivity caused by manual operator gating and (iii) substantially reducing the workload (e.g., extensive staff trainings) and laboratory costs. Recently, several national reference laboratories of the iBFM-FLOW network and the AIEOP-BFM AML FLOW-MRD study group from across Europe [Austria, Germany, Italy, Poland, Russia (Moscow) and South America (Argentina)] have joined forces in a project called flowCLUSTER, dedicated to foster standardization and automation of MFC-MRD analysis in pedAML. They used machine learning technologies (61, 65), similarly to what already pursued in automated MFC-MRD data analysis of acute lymphoblastic leukemiasamples (57, 60, 64). It can be assumed that such an automated tool, together with central review and a program of continuous quality assessment, will provide standardization and high resolution in MFC-MRD assessment.

## Identification and Integration of Suitable LAIPs to Monitor MRD in AML

Diagnostic laboratories have shifted from 4 to 6 colors upto 12 colors MFC, with a substantial improvement of LAIP detection and discrimination between aberrant and normal cells (32, 66). Integration of computational methods into the diagnostic workflow will further facilitate the use of even more complex staining panels. However, suitable markers or patterns of antigen co-expression unequivocally distinct from those of normal hematopoietic cells should be available.

Aberrant LAIPs can be identified at diagnosis in the vast majority of childhood AML cases (16). Nevertheless, LAIPs are not always reliable and sensitive for MRD monitoring due to several reasons (49, 67, 68). First, LAIPs can be expressed only by a subpopulation of AML blast cells, potentially hampering MRD detection in follow-up samples. Second, not all LAIPs are stable during the follow-up, possibly resulting in false-negative MRD estimation (69). Besides, the suitability of an antigen for MRD assessment strongly depends on both the degree of its background expression on normal cells and its discriminative expression pattern.

Current marker panel recommendations, including those published by the ELN (32) contain broadly useful markers like the core/backbone markers CD33, CD34, CD45, CD117, and HLADR, the mandatory markers CD13, CD14, CD15, CD11b, CD38 (32), CD123 (70, 71), CD371 (71, 72), CD45RA (73, 74), and CD99 (70, 75), as well as optional markers as per diagnosis, e.g., CD56. In the recent years several new, promising markers have been identified. CD11a is consistently expressed on normal leukocytes and CD34^+^ progenitors in BM (76, 77). Boztug et al. (54) reported that CD11a deficiency is highly specific for AML-M7, Down Syndrome (DS) AML and transient myeloproliferative disease, making it an ideal candidate for MRD monitoring in these subtypes (Table 2). Advanced technologies including GEP (43) or mass spectrometry based on cell surface capture technology (79) are increasingly used to discover novel markers for MFC-MRD. Recently, Coustan-Smith et al. (43) used genome-wide gene expression analysis to identify different marker profiles specific for AML and normal hematopoietic cells. The authors identified twenty-two markers able to improve the discrimination between leukemic and normal cells. Notably, their expression was stable during chemotherapy, as well as upon relapse (43). In an attempt to identify targets for CAR-T cell therapy, Perna et al. (80) generated an extensive dataset of AML surface proteins using proteomics and transcriptomics. They identified several antigens highly expressed in AML bulk and leukemic stem cells but expressed only at very low levels in normal hematopoietic cells.

**Table 2:** Markers for MFC-MRD in AML.

	**Marker**	**References**	**Comment**
Core markers/Backbone	CD33	(32)	
	CD34	(32)	
	CD45	(32)	
	CD117	(32)	
	HLADR	(32)	
Mandatory markers	CD13	(32)	
	CD14	(32)	
	CD15	(32)	
	CD11b	(32)	
	CD38	(32)	
	CD123	(70, 71)	
	CD371	(71, 72)	
	CD45RA	(73, 74)	
	CD99	(70, 75)	
Optional markers	CD2	(32)	If positive at diagnosis
	CD4	(32)	If positive at diagnosis
	CD7	(32)	If positive at diagnosis
	CD56	(32)	If positive at diagnosis
	CD11a^ⱡ^	(54)	If negative at diagnosis
	NG2^Ⱡ^	(53, 78)	If positive at diagnosis

ⱡ *Usually negative in AML M7 and TMD*.

Ⱡ *Expressed in most cases with 11q23 (KMT2A) abnormalities*.

## May MFC-MRD Represent a Surrogate Endpoint for Survival in Pediatric AML?

In the majority of late-phase clinical trials, the primary endpoints are OS and EFS (81). Hence, a long follow-up period is required before drawing any conclusions on the efficacy of new therapy regimen and new anti-AML-drugs. To accelerate the discovery and approval of new drugs for pedAML, the call for a suitable surrogate for OS and EFS is steadily growing louder. Recently, the Food and Drug Administration (FDA) published a guidance document on the role of MRD in the development of drug products (FDA website, 2018). The use of MFC-MRD as a new early endpoint for the assessment of therapeutic response in clinical trials is intriguing. MFC-MRD detection in AML may expedite drug approval or prevent the adverse continuation of suboptimal treatment strategies (82). Several studies have already shown the potentialities of MRD as surrogate endpoints in AML (83–85).

## Conclusions

With continuous efforts in standardization, MFC-MRD response may guide treatment intensity, and become a useful surrogate endpoint for clinical drug development in pedAML. Hence it is time to perform prospective, multicenter randomized trials to evaluate the impact of therapeutic interventions driven by MFC-MRD, together with its role as an early marker of response-to-therapy and a potential surrogate survival endpoint.

## Author Contributions

BB and MD designed the study. BB, MM-G, EV, and MD wrote the manuscript.

### Conflict of Interest

The authors declare that the research was conducted in the absence of any commercial or financial relationships that could be construed as a potential conflict of interest.
